# Promoting Smartphone Usage Among Older Adults: The Role Of Perceived Emotional Value

**DOI:** 10.1093/geroni/igaf122.3832

**Published:** 2025-12-31

**Authors:** Suyi Wang, Minjie Lu

**Affiliations:** Beijing Normal University, Zhuhai, Guangdong, China (People’s Republic); Beijing Normal University at Zhuhai, Zhuhai, Guangdong, China (People’s Republic)

## Abstract

As society ages and the digital era rapidly evolves, older adults often face challenges in adapting to new technologies. Socioemotional Selectivity Theory (Carstensen et al., 1999) posited that with age, individuals tend to prioritize emotional motivation over instrumental motivation. According to Perceived Value Theory (Zeithaml, 1988), consumers may weigh the value of a product differently, and this could predict purchase decisions (Sweeney & Soutar, 2001). This study examined whether older adults’ prioritization of emotional motivation may make them weigh emotional values (enjoyment or social meanings) of using smartphone over functional values (utilitarian performance). In study 1, 195 participants aged 18 - 80 (108 females, Mage = 45.90, SD = 14.38) reported their social motivations and perceived values of smartphone applications. Results showed that older participants placed greater emphasis on the emotional value of smartphone apps, and this age-related change was mediated by the age-related increase in emotional motivation. Study 2 manipulated the perceived value of smartphone apps through advertising slogans, and 60 younger adults (40 females, Mage = 26.55, SD = 2.87) and 53 older adults (15 females, Mage = 61.20, SD = 2.12) were recruited. Results showed that older adults preferred choosing emotionally appealing apps over functionally appealing apps, but younger adults didn’t show this preference. These findings have practical implications that highlighting the emotional values of smartphone may help promote smartphones usage among older adults. (This project was funded by the National Natural Science Foundation of China, Grant No. 72201037).

